# Global Qualitative Study of Professionals' Perspectives of Caring for People With Head and Neck Cancer Experiencing Suicidal Ideation

**DOI:** 10.1002/pon.70521

**Published:** 2026-06-16

**Authors:** Cherith J. Semple, Kairen McCloy, Sharon L. Bingham, Katherine Penney, David Johnston, Jeffrey R. Hanna

**Affiliations:** ^1^ Ulster University Institute of Nursing and Health Research Belfast UK; ^2^ South Eastern Health and Social Care Trust Ulster Hospital Dundonald UK; ^3^ Northern Health and Social Care Trust Antrim Area Hospital Antrim UK

## Abstract

**Background:**

Globally, people with head and neck cancer (HNC) are twice as likely to die by suicide compared to all other cancers. Yet most HNC suicidality studies focus on estimating prevalence, rather than how to assess and support people with suicidal ideation within routine cancer care. This study aimed to explore professionals' perspectives of how to optimally identify, refer and support people with HNC reporting suicidal ideation.

**Methods:**

Thirty‐nine, semi‐structured interviews were conducted with health and social care professionals caring from people with HNC from 13 countries; and analysed using reflexive thematic analysis.

**Findings:**

Three multilevel influences were identified: (1) patient risk factors: from identity disruption to demoralisation, (2) role ambiguity and emotional burden for professionals, and (3) sociocultural influences relating to suicidal ideation for people with HNC. Despite a divergence in confidence across professional roles, participants acknowledged a need to screen for suicidal ideation, prioritising diagnosis, early post‐treatment, when late‐effects are prevalent (12–24 months), alongside trigger‐based screening. Barriers to implementation include a lack of professional knowledge and confidence, referral pathways and timely access to psychological care. Professionals highlighted the emotional demands and moral injury, identifying the need for suicide prevention training and clinical supervision.

**Conclusion:**

To enhance suicidality management for people with HNC, there is a necessity for clinician‐led needs‐based psychosocial assessment, collaborative safety planning, and clear, stepped‐care referral pathways with integrated psycho‐oncology services. Workforce needs include suicide prevention training and structured clinical supervision. Proactive suicide prevention should be a public health priority given this high‐risk population.

## 1 Background

Globally, over 700,000 people die by suicide each year [1] and 77% of global suicides occur in low‐and‐middle‐income countries (LMIC) [2]. Suicide prevention is a public health priority, with the WHO Sustainable Development Goals aiming to reduce suicide rates by one‐third by 2030 [3]. A cancer diagnosis can trigger a psychological crisis, with reviews identifying that suicide completion rates approximately three times higher among people with cancer compared to the general population [2].

Moreover, patients with head and neck cancer (HNC) are twice as likely to die from suicide compared to other cancer populations [4, 5]. Evidence suggests that suicidality in HNC is more prevalent among males, older adults (≥ 65 years), those who are widowed or divorced, and individuals with hypopharyngeal cancer or poorly managed symptoms [6]. To reduce preventable deaths in this high‐risk population, suicide prevention is of paramount importance for patients with HNC. Globally, an estimated 6.8% of people with HNC experience suicidal ideation, defined as thoughts of self‐harm or suicide [7].

Bronfenbrenner's (1979) [8] socioecological framework provides a useful lens for understanding suicidal ideation in HNC, emphasising how wellbeing is shaped by interacting personal, relational, healthcare and societal systems [9, 10]. Contemporary research addressing suicidal ideation for people with HNC has predominately focussed on associations with sociodemographic and clinical parameters [6], with little attention to the complex interplay of psychological variables or alignment to suicide theories within cancer care [11, 12]. Conceptualising suicidal ideation for people with HNC as multifactorial enables the exploration of both risk and protective factors, and the complex interdependence of biopsychosocial factors [11].

Health and social care professionals (*professionals*) are well positioned to identify, refer, assess and support people with HNC who experience suicidal ideation [6, 12]. Actively engaging patients in conversations about suicidality has proved beneficial [13], especially as patients often do not readily disclose suicidal ideation due to shame, internalised stigma or influences of conformality with traditionally masculine norms [14]. Despite these insights, research indicates that recognising risk, openly discussing suicidal thoughts and intervening is inadequately addressed in routine cancer care [15]. Risk identification remains inconsistent, with evidence from systematic reviews highlighting that suicide risk assessment tools are poor at reliably predicting suicide [12, 16, 17]. Also, screening tools are often considered too general, burdensome or insensitive to the unique risks associated with HNC [12, 16, 17]. Furthermore, managing suicidal ideation in clinical practice is frequently described as emotionally complex and challenging; with professionals reporting limited confidence and knowledge, feelings of guilt, distress and uncertainty regarding professional roles and responsibilities [18].

While suicidal ideation has been explored in cancer populations more broadly, research focussing specifically on HNC remains limited [13, 17]. Given the heightened risk within this group and the crucial role of professionals in suicide prevention, there is a need to better understand how professionals perceive and navigate the complexities of identifying and managing suicidal ideation for people with HNC. Ultimately, this could better inform patient screening and clinical interventions for people with HNC experiencing suicidal ideation [19] and inform targeted education for professionals to improve care for this vulnerable population [20].

### 1.1 Aim

To explore professionals' perspectives of how to identify, assess, manage and support people living with and beyond HNC who are experiencing suicidal ideation.

### 1.2 Objectives

1. Explore professionals' experiences of providing care to people with HNC experiencing suicidal ideation,
2. Investigate professionals' perceptions of the risk and protective factors for suicidal ideation in people living with and beyond HNC,
3. Explore professionals perceived role in identifying, assessing, managing and supporting people living with and beyond HNC who are experiencing suicidal ideation,
4. Identify professionals perceived strategies for optimally supporting individuals with HNC experiencing suicidal ideation.

## 2 Methods

### 2.1 Study Design

A descriptive qualitative design using semi‐structured, one‐to‐one interviews [21], informed by Bronfenbrenner's (1979) [8] socioecological framework was employed. Closely aligned with this study's aim, a descriptive qualitative design provided an epistemologically coherent and methodologically pragmatic means of capturing the diverse, nuanced, and context‐bound realities of global HNC professionals' experience of managing suicidal ideation. This enabled participants to share their experience and describe practices, perceptions and complexities within their clinical setting [21]. Semi‐structured, one‐to‐one interviews were chosen because suicidality in HNC represents a highly sensitive and emotionally complex area of practice. One‐to‐one interviews offered the privacy, psychological safety, and depth required for participants to openly discuss emotionally challenging clinical experiences, while allowing flexibility to explore diverse clinical contexts and professional perspectives [21]. This method also mirrors the relational dynamics through which suicidal ideation is typically disclosed in real‐world HNC care, enhancing conceptual coherence between the phenomenon and method. One‐to‐interviews also avoided issues with group coordination, challenges with scheduling across time zones and equitable regardless of role seniority and location. Reporting followed the Consolidated Criteria for Reporting Qualitative Research (COREQ) guidelines [22].

### 2.2 Study Population

Study population was registered professionals (i.e., doctor, nurse, therapeutic counsellor, social worker, allied health professional, paramedic), involved in the care of people with HNC, across hospital or community settings. For eligibility, there were no limits applied to professional disciple, nor years of professional experience or geographical location. The only exclusion criteria were lack of a professional registration and the inability to speak and write in English.

### 2.3 Sampling

Purposive sampling was used to recruit a diverse range of professionals, from across the globe.

### 2.4 Recruitment Procedures

A flyer (Supplementary Material 1) with brief study details was developed by members of the research team (CJS, JRH) and disseminated via professional networks, social media platforms (LinkedIn), and newsletters of relevant cancer organisations between November 2024 and January 2025. Embedded within the study flyer was contact details for the research team enabling potential participants to ask questions before participating and an electronic link to a participant information sheet and consent form, operated via JISC survey software. Individuals provided their email address within the consent form, enabling online one‐to‐one interviews to be scheduled via MS Teams at a mutually convenient time.

### 2.5 Data Collection

Prior to the interview, participants completed an online sociodemographic questionnaire capturing details surrounding their role, years of professional experience, and personal experiences of suicidal ideation or bereavement by suicide. Semi‐structured interviews were conducted between December 2024 and March 2025 using an iteratively refined topic guide informed by the literature, Bronfenbrenner's socioecological framework, clinicians, researchers and patient and public involvement (Supplementary Material 2). Bronfenbrenner's socioecological framework [8] was a sensitising guide for topic guide development, but data was collected grounded in participants' accounts rather than predefined assumptions.

Interviews were conducted by two experienced clinical‐academic cancer nurse researchers (CJS [48%], JRH [52%]) with no previous personal relationship with any participants, lasting between 16.25 and 58.03 min (*mAvg* = 35.84 min). Reflective field notes were completed by the interviewer (CJS, JRH) after each interview to support analytic reflexivity and discussed at research team meetings. Interviews continued until no new categories were identified and a meaningful global sample was achieved.

### 2.6 Data Analysis

Audio‐recordings from MS Teams were professionally transcribed verbatim and managed using NVivo v14. Data were analysed using Braun and Clarke's reflexive thematic analysis [23], following an inductive, iterative process (familiarisation, open coding, theme development) with researcher reflexivity central to theme construction. This approach provided analytic flexibility consistent with our descriptive qualitative design, which enabled rich, inductive data‐driven theme development across a global sample centred on participants' accounts.

Initially, CJS repeatedly read transcripts to achieve familiarisation, followed by inductive line‐by‐line scrutiny and independently coding each transcript. Deploying an inductive method, codes were organised into meaningful clusters and formed a mind map with input from team members (JRH, KMcC), from which themes were collaboratively developed and refined by the research team. The detailed reflections constructed after each interview (CJS, JRH) helped inform the themes. Final themes were discussed and verified with all authors.

### 2.7 Patient and Public Involvement and Engagement (PPIE)

Patients with lived experience of HNC (*n* = 6), clinicians (*n* = 2) and researchers (*n* = 2) helped develop the topic guide. Study findings were reviewed by patients with lived experience of HNC (*n* = 4), a clinical psychologist (*n* = 1), and psychiatrists (*n* = 2) and their insights helped refine the clinical recommendations.

### 2.8 Ethical Considerations

Ethical approval was obtained by the Ulster University Institute of Nursing and Health Research Ethics Filter Committee (FC‐NUR‐24–096). Participants provided written informed consent and verbal consent prior to interviews. The right to withdraw was emphasised, and participants were offered debriefing and signposting to support (also included in participant information sheet) if interviews raised distress. Awareness of participants' lived family experience of bereavement by suicide or suicidality, to include personal suicidal ideation informed sensitive interview conduct.

## 3 Results

Thirty‐nine professionals were recruited from 13 different countries. Sample characteristics are reported in Table 1.

**TABLE 1:** Sample characteristics.

	*n* (39)
Professional role	
HNC clinical nurse specialist	10
HNC dietitian	2
HNC speech and language therapist	5
HNC surgeon	2
HNC oncologist	2
HNC psychologist	2
General practitioner (GP)	3
Specialist radiographer	4
Oncology counsellor	1
Social worker	1
Paramedic	1
HNC SHO	1
Palliative medicine registrar	1
Palliative medicine consultant	1
Occupational therapist	1
Oncology mental health nurse	1
Dentist	1
Job setting	
National healthcare organisation	34
Private healthcare organisation	4
Charitable organisation	1
Formal training on suicide prevention	
Yes	12
Mandatory training (*n* = 3)	
Clinical training programme (*n* = 5)	
Postgraduate studies (*n* = 2)	
eLearning (*n* = 2)	
No	27
Personal experience of death by suicide	
Yes	12
Friend (n = 9)	
Immediate family member (n = 3)	
No	27
Location of participant	
UK (England, Scotland, Wales, NI)	20
Republic of Ireland	3
Portugal	2
India	2
Australia	2
Singapore	2
USA	2
France	1
Denmark	1
Sweden	1
Canada	1
Zimbabwe	1
Taiwan	1
Years professional experience	
0–5 years	14
6–10 years	7
11–15 years	10
16–20 years	4
21–25 years	0
36–30 years	3
31 years+	1
Years professional experience in HNC	
0–5 years	7
6–10 years	10
11–15 years	10
16–20 years	6
21–25 years	4
36–30 years	2
Gender	
Male	8
Female	31

**FIGURE 1:**
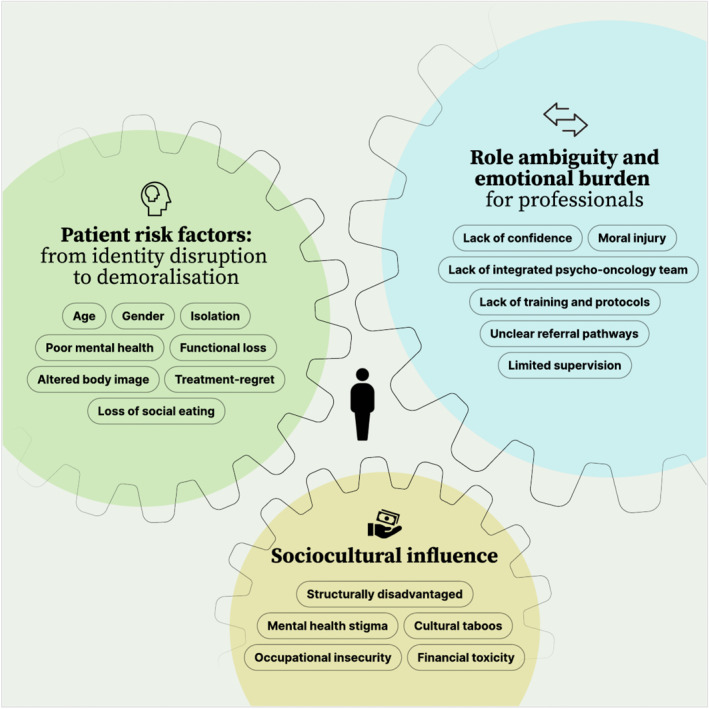
Multilevel factors influencing suicidal ideation for people with head and neck cancer.

Salient findings were often replicated irrespective of geographical location of the participants. Data highlighted interconnected disease‐related, social and psychological complexities for people with HNC experiencing suicidal ideation, which are displayed in Figure 1, alongside multilevel professionals and societal factors. The risk and protective factors are reported across three themes are: (1) patient risk factors: from identity disruption to demoralisation, (2) role ambiguity and emotional burden, and (3) sociocultural influences relating to suicidal ideation for people with HNC.

Theme 1 Patient risk factors: from identity disruption to demoralisation.

Most professionals described *gender and age‐related* risk factors among people with HNC expressing suicidal ideation as ‘*nearly always gentlemen, nearly always 60s or older, those in their 70s even more so*’ (P1, SLT). Additional concerns were voiced for a subgroup of males who were younger, often with HPV‐related cancer and described as pre‐morbidly highly‐functioning, experiencing abrupt everyday disruptions from their treatment. One participant observed, ‘*For this younger person everything had changed… work, dating, going to the gym…*’ (P17, Radiotherapist). Females were rarely mentioned, reinforcing perceptions of a predominantly male risk profile. Furthermore, it was perceived that men were ‘more likely to act’ compared to women, who were seen as more open to psychological intervention when experiencing suicidal ideation.

*Social isolation* was repeatedly highlighted as a critical driver for suicidal ideation, coined as ‘loneliness is a big killer’ (P2, CNS). Participants described unmarried, bereaved or divorced men with limited networks who ‘*don't go out as much and become more socially isolated as a result of the cancer treatment*’ (P3, Dietitian) to be at higher risk of suicidal ideation. Also, those with *premorbid mental health issues* including depression, anxiety, substance misuse histories, post‐traumatic stress disorder and experiences of trauma (including military service) were reported as having an amplified risk, especially when coupled with loss of independence.

Across the findings, suicidal ideation in HNC emerged less as a discrete mental health event and more as an endpoint of a *multi‐factorial cascade of identity disruption*, which can lead to a loss of connectedness and participation in valued roles (e.g., singers, lecturers, parents); often framed as *demoralising* rather than explicit suicidal intent. This sense of demoralisation was complex and precipitated not only loss of social participation and loneliness but interconnected with persistent treatment‐related functional losses (speech and swallow), changes to body image, pain and symptom clusters (dysphagia, xerostomia, trismus, fatigue), erosion of work and family roles and financial strain. Demoralisation from loss of confidence when eating and drinking with others was especially profound, emerging as a key risk factor to suicidal ideation. Most professionals observed that compounded by limited adaptable dining options for people with HNC, leaving them excluded from routine social events. This was often punctuated by entrapment from *treatment‐regret* and a sense that ‘*life will never be the same….I just wish I wasn't here; I wish I hadn't done it.*’ (P26 SLT). Inadequate preparation for the sequalae of HNC treatment significant contributed to people with HNC having unrealistic expectations for their everyday life post‐treatment, which was perceived as a driver of distress and treatment regret. One professional noted, ‘*One of the things that catches them off guard is that it is worse than they think it's going to be*’ (P1 SLT), underestimating the intensity and duration, leading to shock and frustration. Some professionals considered patients as feeling pressured into extensive surgical and adjuvant plans without fully understanding the life‐changing implications: ‘*We see a lot of treatment regret… people feel pressured into doing things which maybe is a problem*’ (P2, CNS).

Most professionals felt the greatest risk for suicidal ideation was early post‐treatment (3–6 months). This was often related to fewer appointments with the specialist HNC team, or inherent anxiety associated with post‐treatment scans, plus diminishing social support when late‐effect and demoralisation peaks. The other high‐risk window considered for suicidal ideation was later post‐treatment (after 1–2 years) when undesirable late effects persisted diminishing quality of life, or when recurrence happen: ‘*like being 5 years, or 7 years down the road, to undergo treatment again. That also puts them at risk again.*’ (P24, Psycho‐Oncologist).

Theme 2 Role ambiguity and emotional burden for professionals.

It was believed that people with HNC disclosed suicidal ideation more readily to clinical nurse specialists and allied health professional, rather than to surgeons/oncologists, due to factors such as relational continuity and perception of having more time: ‘*For some it might be the relationship with their specialist nurse… often that's the person they see frequently*’ (P15, Psychology Counsellor). Similarly, a speech and language therapist noted that one‐to‐one settings facilitated openness: ‘*We spend more time with patients… more confident asking questions… patients tend to open up to us*’ (P25, SLT). Most consultants acknowledged they rarely asked directly about suicide ideation in oncology clinics.

Apart from teams who had integrated HNC psycho‐oncology service provision, most professionals described uncertainty about their role and remit when supporting people with HNC experiencing suicidal ideation: ‘*I really struggle to know what my role is and where to go with it… we're not really trained in that area*’ (P1, SLT). Many professionals, across nursing, medicine and allied health disciplines expressed a fear of ‘*opening up a can of worms*’ when services are limited and ‘*what to do if patient declines help*’ (P11, CNS). Consequently, the *emotional burden* and moral injury of managing suicidal ideation was profound. Professionals reported guilt, fear, and fatigue from ‘sitting in the discomfort’ of such harrowing conversations. Consequently, ‘*You definitely take it home with you… emotionally it can be quite fatiguing*’ (P11, CNS). Others described feeling like failures: ‘*You leave feeling quite disheartened that you haven't done a good job*’ (P3, Dietitian). One participant framed this as ‘*part of the moral injury in medicine… we are aware of this problem, and we don't have a lot of solutions*’ (P23, Dentist). This was compounded by limited clinical supervision ‘*We don't get any clinical supervision, I don't want clinical supervision from a Band 5 nurse*’ (P16, Radiotherapist) or inadequate restorative support: ‘*I had three patients who committed suicide within a year who I was looking after … there's no support really so you just get on with it*’ (P18, GP). Where supervision and multidisciplinary debriefing existed, more commonly within teams were psychological services where embedded, these support mechanisms were highly valued for resilience‐building: ‘*I feel like it's part of our profession to have lots of supervision. I'm always thankful for that as it doesn't feel like you're handling it alone*’ (P24 Clinical Psychologist). Many professionals also reported limited training opportunities in suicide prevention, noting they were often ‘grabbing in the dark’ (P11, CNS). More often, participants indicated that simulation‐based and case‐driven training should be prioritised to build confidence and self‐efficacy, with instructional eLearning training deemed insufficient, ‘*Watching a video… doesn't do anything*’ (P17, Radiotherapist).

**FIGURE 2:**
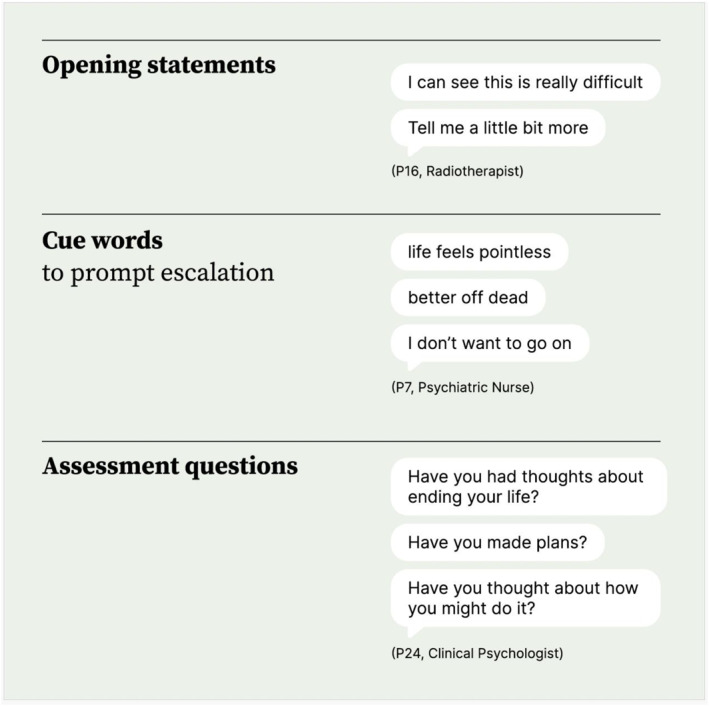
Language used to aid screening and assessment of suicidal ideation.

Participants highlighted significant *system‐level gaps and barriers* when supporting people with HNC experiencing suicidal ideation. To help identify suicidal ideation, validated tools were infrequently used, with some professionals acknowledging an awareness of local instruments but ‘*uncertain about how to apply it*’ (P14, CNS). Few participants reported using generic validated tools such as the PHQ‐9 and GAD‐7 but importantly viewed them as merely prompts for structured conversations and safety planning conversations. There was a strong consensus that suicide risk assessment tools with ‘tick‐boxes’, deriving output scores should not replace a clinical psychosocial assessment; and were considered as poor at preventing or predicting suicide and deemed unreliable decision aids. General practitioners within this study emphasised that confidence in asking difficult questions comes with experience, after having formal training: ‘*It's hard to replicate experience… being prepared to ask difficult questions comes with time*’ (P30, GP). Some examples of language used during structured conversation by professionals aiding assessment of suicidal ideation can be found in Figure 2.

When pathway ambiguity was present for people with HNC at imminent risk of suicide, professionals referred patients to Emergency Departments for urgent mental health assessments. This was deemed necessary but suboptimal for managing a mental health crisis in HNC care, described as ‘*even more distressing…good practice would not be sending people to A&E*’ (P19, Oncologist). Also, mental health crisis services were often described as slow, overwhelmed and sometimes perceived as inconsistent with referrers risk judgements.

Conversely, having protocols and suicide care pathways with clear escalation steps provided professionals with clarity and confidence: ‘*It's kind of like a flow chart that we follow… low risk gets a call back in 2–4 weeks… high risk, psychiatrist calls same day*’ (P7, Psychiatric Nurse). Rapid access to specialist advice was a critical enabler. Even when face‐to‐face psychiatry was unavailable, telephone support was valued: ‘*If very high risk… we've been in contact with psychiatry and got advice… they've been really helpful*’ (P6, Palliative Medicine). An integrated psychological and liaison psychiatry service was seen as a protective factor, promoting a HNC team‐based approach to suicidal ideation management. Some professionals considered this approach as transformative; enabling needs‐based psychosocial assessment, creating safety netting for patients and empowering professionals to act decisively and compassionately ‘*Since we've had our psychologist team in place… things have been a lot easier*’ (P16, Radiotherapist). However, it was clear there was unequal access and varied responsiveness from psychological services, for example ‘*If we refer to psychology it might take 12 weeks, which is way too late*’ (P1, SLT). Others experienced referrals rejected with patients not meeting the strict ‘risk’ criteria, or geographical inequity with some centres having team‐based psychologists, whereas others relied on external services and some none at all.

Theme 3 Sociocultural influences relating to suicidal ideation for people with HNC.

Societal and cultural factors presented as profound determinants of how suicidality in HNC is disclosed, perceived and managed. Mental health stigma regarding suicide and cultural taboos were repeatedly highlighted as pervasive, particularly among older generations ‘*who find it easier to talk about the somatisation of their symptoms rather than the emotional symptoms that they're experiencing*’ (P24, Clinical Psychologist) and within certain cultural contexts such as South Asia were mental help‐seeking viewed as shameful: ‘*Mental health doesn't exist—you don't talk about it [in my community].*’ (P16, Radiotherapist). Participants recognised that this reluctance, coupled with fear of being judged, reinforced barriers to care and underscored the need for culturally sensitive suicidal ideation communication strategies and proactive normalisation of mental health care within oncology pathways.

Socioeconomic strain further compounds risk, with financial insecurity and job loss described as catalysts for suicidal ideation: ‘*If we correct the income… the suicidal thought could be eliminated*’ (P29, SLT). Structural disadvantages included those HNC patients without welfare benefits or with precarious work contracts. For insurance‐based healthcare systems, out‐of‐pocket cost was reported as impeding mental‐health uptake. Furthermore, occupational stigma, particularly amongst older people with fear of career repercussions and employment jeopardy, reinforced silence and isolation, further exacerbating risk.

## 4 Discussion

This global study identified complex and interconnected risk factors relating to suicidal ideation in people with HNC, linking social and psychological dimensions closely with the illness itself, and treatment‐related effects. In addition to the emotional toil for professionals caring for this high‐risk patient population, patient and professional issues are coupled with a lack of system‐level structures for assessment and referral pathways and integration of psycho‐oncology services within HNC teams. Public stigma surrounding suicide ideation, with nuanced cultural taboos regarding mental health reinforces a lack of disclosure, increasing risk of suicidality in HNC.

Consistent with previous psycho‐oncology and HNC literature, gendered and age‐related risk patterns were concentrated among men, particularly those aged 60+ [6]. However, in keeping with the changing HNC landscape depicting an increased prevalence of younger men with HPV‐related HNC, findings reported that younger men whose work, appearance, and social roles were abruptly disrupted by treatment were also at risk of suicidal ideation. This reinforces the concept of ‘masculinised vulnerability’, where gender norms for males frequently link identity to stoicism, productivity and autonomy, making functional loss and disfigurement particularly destabilising, heightening suicide risk and inhibits help‐seeking [14, 24]. Importantly, men are less likely to verbalise distress and more likely to act, underscoring the need for proactive screening rather than reliance on patient disclosure. Therefore, from a suicide prevention perspective, this pattern reflects well‐established evidence that male suicide risk is often mediated less through expressed affective distress and more through role threat, thwarted belonging, and perceived loss of agency, necessitating gender‐responsive identification strategies [25].

There is also a need to mediate, honest, clear and realistic pre‐treatment information, alongside professional relational continuity for suicide prevention in cancer care [26]. Interventions should prioritise continuity of psychosocial support beyond active treatment, with targeted strategies for older men living alone and younger men experiencing role loss. Future research should explore culturally sensitive approaches to engage men in psychological care and evaluate models that combine symptom management with social reintegration to mitigate isolation‐driven risk.

Consistent with conceptual theories in cancer and suicidality [12] and highlighted within the global literature, intersecting economic and relational losses may erode protective buffers overtime, increasing vulnerability particularly in the absence of compensatory or institutional supports. Therefore, with financial insecurity and job loss described as catalysts for suicidal ideation [7]; this indicates a need to embed financial support early [27]. Additionally, social isolation appears to be a critical mechanism for risk, amplified by bereavement of spouse/partner, pre‐existing lifestyle factors such as alcohol and tobacco use, or treatment‐related functional loss relating to eating and speaking, narrowing opportunities for socialising [28].

The early post‐treatment phase was identified as a high‐risk period for suicidal ideation; heightened by reduced clinical contact and treatment‐related symptom clusters. These intertwining factors appear to impact self‐identity, often regarded as demoralisation with expressions of treatment‐regret. Importantly, demoralisation in this context should be distinguished from depressive disorder, as suicidology literature consistently demonstrates that feelings of helplessness, entrapment, and loss of meaning can independently confer suicide risk, even in the absence of depression [29].

It appeared that clinician‐based identification of suicidal ideation largely relied on impromptu judgement, rather than structured protocols or formal suicide‐specific instruments or structured conversations applied longitudinally. This reliance on informal judgement mirrors broader critiques within suicide research, which consequently perpetuates inequities in suicidal ideation recognition and response; rather than skilling professionals in structured, culturally sensitive team‐based suicide prevention strategies [30, 31]. This underscores the need to collaboratively identify protective factors, immediate supports, and means‐restriction, which are explicitly link to safety planning rather than risk scoring [32]. These systemic barriers such as a lack of structured protocols and clinical pathways, limited integration of psych‐oncology services, coupled with lack of suicide‐specific training opportunities contributed to role ambiguity and moral distress for professionals, which aligns with recent global studies [5].

Societal and cultural factors were identified to have a profound influence on suicidal ideation in HNC, shaping both patient disclosure and clinician response. Across studies globally, cultural norms influence not only suicide disclosure but also how distress is conceptualised and legitimised within healthcare encounters [33]. Addressing societal drivers, such as pervasive mental health stigma, especially amongst certain cultural contexts, requires culturally adapted multi‐level suicide prevention interventions in oncology settings [34]. This should include public health campaigns normalising mental health dialog within oncology care through culturally sensitive communication.

### 4.1 Strengths and Limitations

Findings are reflective of a range of professionals caring for people with HNC experiencing suicidal ideation, across various settings (primary and secondary care), from 13 countries. However, capturing the voice of patients and family caregivers is outside the scope of this paper but will importantly inform subsequent planned studies. Although this study addresses a gap in the literature, limitations to this study include a self‐selected population, with higher personal and professional experience of death by suicide and less professionals recruited from LMICs. It is possible, with under‐representation of professionals from LMIC countries, who have the highest rate of suicide, that suicide prevention strategies in these HNC settings may need additional consideration of cultural and societal issues to mediate and moderate for the transition from suicidal ideation to suicidal acts.

### 4.2 Clinical Implications

There is a need for a multi‐layered approach to managing suicidality in HNC, combining structural, relational, and educational strategies to shift care from reactive crisis management towards proactive, culturally attuned prevention. Informed by study findings, existing literature and refine through PPIE input recommendations for suicide risk mitigation in HNC and workforce support are presented in Table 2.

**TABLE 2:** Clinical recommendations for suicide risk mitigation and workforce support when managing people with HNC.

Clinical recommendation	Implementation strategies
Clear, realistic pre‐treatment information	*Early, honest pre‐treatment counselling* about the severity, persistence and uncertainty of side‐effects (e.g. mucositis, dry mouth, swallowing and speech difficulties, altered appearance) before major interventions. This should include discussion of *social impact*, including eating and drinking in public, to prepare patients for lifestyle changes and reduce treatment‐regret. Offer written summaries of key pre‐treatment discussions and or facilitate a peer‐visit with a similarly matched patient who has completed treatment. *Optimise shared decision‐making* during the pre‐treatment period to avoid perceived pressure and empower patient choice. *Revisit pre‐treatment discussions after treatment* to help patients contextualise what they are experiencing and reduce self‐blame or regret.
Proactive screening, assessment and safety planning	Incorporate routine *screening* of mood and suicidal ideation at *multiple time points* when risk of suicidal ideation is likely to be greatest: Pre‐treatment, post‐treatment (especially 3–6 months after completion, then 12–24 months) and at recurrence to identify possible current suicide risk. Include trigger‐based screening (not solely time‐point screening), for example: ◦ Significant functional deterioration (e.g. feeding tube insertion, tracheostomy changes) ◦ Hospital admissions or repeated A&E attendances ◦ Major social stressors such as job loss or bereavement. Of note, the purpose of screening is to detect current risk, not to predict future suicide. For clinical *assessment* use *structured, clinician‐led compassionate person‐centred assessment* approaches rather than risk assessment tools to understand: ◦ Presence, frequency, and intensity of suicidal thoughts ◦ Intent, planning, and preparatory behaviours ◦ Time course, triggers, and recent changes ◦ Psychological, social, and medical contributors ◦ Protective factors and sources of support ◦ Access to lethal means and feasibility of restriction.
Target psychological interventions for patients with high‐risk factors	Prioritise *men* who are *isolated* and those *experiencing identity and role disruption.* Pay special attention to patients who are *unmarried, divorced, bereaved*, or *living alone, experiencing treatment‐related functional loss* and *disfigurement,* have *pre‐existing mental health* issues to include alcohol and substance misuse and PTSD. Intersectionality can often compound risk.
Address social isolation	Facilitate *peer support groups*, buddy systems, and community‐based interventions. Explore *supported social eating interventions* and communication aids to restore social participation.
Integrate psycho‐oncology and mental health liaison services within teams	Funding for *dedicated psycho‐oncology posts* within HNC multidisciplinary teams to ensure availability and timely access. Create *rapid referral pathways* with mental health liaison services for high‐risk patient reducing reliance on emergency departments. Expand *telehealth psychological support* to address geographic inequities.
Manage functional loss, symptom burden and address psychosocial issues.	*Proactively treat and manage symptoms* including pain, mucositis, fatigue, lymphoedema, body image distress, sexual functioning and intimacy difficulties and chronic sleep disturbance. Provide *early rehabilitation* for speech, swallowing and range of neck and shoulder movements. Include *vocational counselling* to address return‐to‐work challenges. Address *financial constraints* through welfare support and insurance navigation.
Reduce barriers to access of psychological services	Offer *male‐sensitive engagement strategies* (e.g., practical problem‐solving, normalising distress) to overcome reluctance to seek help. Provide *flexible appointment formats* (telehealth) to combat appointment fatigue. Include *assertive outreach* models for patients who decline or disengage from psychological support as lack of engagement does not equate to ‘low risk’.
Workforce training	*Mandatory introductory suicide awareness training* for all HNC multidisciplinary team (MDT). Develop *tiered psychological skills training,* with intermediate level training for professionals responsible for delivering frontline care, to enable safe initial conversations to identify and assess risk, incorporating: ◦ *Simulation‐based learning* and role‐play for real‐world scenarios on suicide risk conversations and crisis response. ◦ *Case‐based reflective sessions* to build comfort with ‘sitting in the discomfort’ rather than rushing to offer solutions to ‘fix’ problems, managing uncertainty when risk is ambiguous. Within *training include moral injury and cumulative exposure*, particularly for professionals with long‐term relational responsibility for people with HNC.
Staff support and clinical supervision	Protected time for *structured, restorative clinical supervision* for all MDT members. Introduce *peer‐debriefing* and *resilience workshops* to mitigate burnout and moral distress. Access to *psychological support for staff* following patient suicide or serious self‐harm, including structured post‐incident debriefs.
Develop clinical pathways with escalation protocol	Implement *standardised escalation protocols* for suicidal ideation: ◦ Establish *rapid referral protocols* to crisis mental health teams with same‐day psychiatric review for high‐risk cases. *Clearly document crisis team contact details* and *GP involvement* in healthcare records, accessible to all relevant services. Ensure *continuity of psychosocial care* beyond active treatment, with primary care having ongoing psychosocial input once patients are discharged from specialist HNC follow‐up.
Systemic and policy‐level actions	Advocate for *national guidelines* on suicidality management in head and neck oncology. Secure *ring‐fenced funding* for mental health integration in cancer pathways. Incorporate *social work and welfare support* to address *social determinants*: Automatic social‐work referral for at‐risk profiles (loneliness, job loss, benefits advice), facilitated re‐entry to work where feasible, and access to community resources through social prescribing. Improve *communication between primary and secondary care* to prevent gaps in follow‐up.

## 5 Conclusion

The study provides rich granularity to the complex, interconnected biopsychosocial risk factors for suicidal ideation in HNC. To mitigate suicide risk for people with HNC, system‐level and societal reforms are necessary. System‐level priorities include embedding psychological expertise within HNC services, developing clear escalation protocols and referral pathways, providing workforce training and supervision to mitigate emotional burden and improve patient safety. Addressing societal drivers requires embedding social support, tailoring gender‐responsive care, and implementing public health strategies to dismantle stigma. Without such systemic and cultural shifts, clinical efforts risk remaining reactive rather than preventive.

## Funding

The study was funded by South Eastern Health and Social Care Trust, Northern Ireland, United Kingdom and awarded to CJS and JRH. The funders had no role in study design, data collection and analysis, decision to publish, or preparation of the manuscript.

## Conflicts of Interest

The authors declare no conflicts of interest.

## Supporting information


Supporting Information S1

## Data Availability

All data relevant to the study are available on reasonable request from the corresponding author.
