# Supplementary material for: Global Qualitative Study of Professionals' Perspectives of Caring for People With Head and Neck Cancer Experiencing Suicidal Ideation
Source: Psychooncology. 2026 Jun 16;35(6):e70521. doi: 10.1002/pon.70521 (PMC13272928; doi:10.1002/pon.70521)
Supplement: Supplementary file 1 — Supporting Information S1 [file PON-35-e70521-s001.docx]

Research opportunity for healthcare professionals
_________________________________________________________________________________________**Suicide** and

**head and neck cancer**
_______________________________________________________________________________________**WHY:** To explore healthcare professionals’ views on assessing and managing patients living with and beyond head & neck cancer with suicide ideation.

**WHO:** Healthcare professionals directly involved in caring for people with head and neck cancer.

**WHAT:** One-to-one online interview lasting approximately 40-minutes.

**When:** At a time suitable and convenient for you.

**HOW:** Scan the QR code for more information or contact:

**Prof Cherith Semple: c.semple@ulster.ac.uk**
